# Dolutegravir Plus Lamivudine Two-Drug Regimen: Safety, Efficacy and Diagnostic Considerations for Its Use in Real-Life Clinical Practice—A Refined Approach in the COVID-19 Era

**DOI:** 10.3390/diagnostics11050809

**Published:** 2021-04-29

**Authors:** Valeria Cento, Carlo Federico Perno

**Affiliations:** 1 Department of Oncology and Haemato-Oncology, University of Milan, 20122 Milan, Italy; valeria.cento@unimi.it; 2 Department of Diagnostic and Laboratory Medicine, IRCCS Children Hospital Bambino Gesu’, 00165 Rome, Italy

**Keywords:** dual therapy, integrase strand transfer inhibitor, treatment simplification, drug-naïve, HIV drug-resistance, archived resistance

## Abstract

The diagnostic and therapeutic management of the Coronavirus Disease 2019 (COVID-19) pandemic in the HIV population brought some known criticalities (and opportunities) to the forefront, for both those who are facing their first therapeutic line today, and for those already well viro-suppressed. The clinical, socioeconomic, and psychological impact of the COVID-19 pandemic should not affect the long-term care of people living with HIV, which creates an urgent need to optimize the diagnostic and treatment approach to the first-line or switch regimens. The use of dolutegravir plus a lamivudine two-drug regimen is one of the most promising solutions to ease the management of HIV treatment in this difficult period. In this review, we report the most salient features related to the use of this regimen from real-life cohorts, meta-analyses, randomized clinical trials, and studies presented at international conferences up to March 2021. We focused on the diagnostic and clinical-management implications of its use in real life, and how these comply with the contingent historical situation. The issue of the timing and type of diagnostic procedures and the relevance of classical diagnostic tests (such as genotype for resistance detection) is also discussed. According to the currently available results, dolutegravir plus a lamivudine two-drug regimen represents an outstanding tool, whose expected advantages fulfill the current requirements for optimal daily care of our HIV patients.

## 1. Introduction

Over the last year, the HIV treatment scenario has required revision and reassessment, in a way that we could not expect. It was not only the new scientific evidence on the efficacy, safety and manageability of the most recent regimens that required this intervention. The management of the Coronavirus Disease 2019 (COVID-19) pandemic in the HIV population further highlighted some critical issues (and opportunities) that emerged independent of COVID-19 (but were increased by its presence), both for those facing their first therapeutic line today, and for those who are already effectively viro-suppressed. The COVID-19 pandemic created the need for a change in our common recommendations for combination antiretroviral treatment (cART), to fulfill the need to simplify patient visits for diagnosis, medications and tests. This necessitated the use of a simple, well-tolerated regimen with high efficacy, and a high barrier to resistance [1]. This raised the question regarding the opportunity to maintain the diagnostic algorithm, as done previously; this is true in the case of the genotypic test for the detection of resistance to antiretroviral drugs, for the assessment of CD4 test in clinical practice, as well as for the timing of the performance of viral load test (every 4–6 months? Yearly? Less frequently?), as with HIV-RNA, but also with attention to HIV-DNA that, in patients with suppressed viral load, may have a relevant clinical role.

International recommendations, such as those regularly updated by British HIV Association (BHIVA) and European AIDS Clinical Society (EACS), stated the preferential use of “high barrier regimens and non-tenofovir (TDF) regimens”, to reduce the frequency of monitoring, and of regimens with few major drug–drug interactions (thereby excluding protease inhibitors, which are boosted agents), ideally in single-tablet regimens without food requirements [1].

Integrase strand transfer inhibitor (InSTI)-based cART is a highly potent, cost-effective and safe combination, recommended for treatment of both drug-naïve and treatment-experienced individuals infected with HIV-1 [2,3,4]. Among available InSTI, dolutegravir (DTG) is currently the most commonly used option for either initiating or switching cART [5,6].

The use of DTG-containing two-drug regimens (2DR), and, in particular, of DTG plus lamivudine (3TC), is one of the most promising solutions to the need to ease the management of HIV treatment without harming its efficacy and safety. In this review, we report on the most salient features of DTG plus 3TC 2DR, with a particular focus on the diagnostic and clinical-management implications of its use in real life, and in the contingent historical situation.

## 2. Diagnostic Framework for Dolutegravir Plus Lamivudine Two-Drug Regimens Use in cART-Naïve Patients

DTG/3TC, is a single-pill, once daily (independent from meals) 2DR regimen, approved by both the US Food and Drug Administration (FDA) and the European Medicines Agency (EMA) [7], and increasingly recommended by the latest versions of international guidelines (Table 1).

Some of the features associated with its use, both in current clinical practice and in randomized clinical trials (RCTs), allow us to outline the most appropriate diagnostic and monitoring scenario in the short- and long-term, and to optimize its versatility in the COVID-19 pandemic setting.

### 2.1. Baseline Laboratory Evaluations

Several laboratory tests are important for the initial treatment of people with HIV, and some of them should be performed after the initiation or modification of cART to assess their efficacy and monitor for laboratory abnormalities that may be associated with toxicity.

The current recommended laboratory assessments for monitoring HIV patients beginning a first-line regimen with DTG+3TC are consistent with those used for cART, as shown in Table 2 [2,4].

An important aspect to consider with this 2DR is the need to assess the initial VL, and confirm the absence of HBV co-infection. In case the patient is HBV-infected (positive HBsAg and/or HBV-DNA test result), a 3DR-containing TDF or tenofovir alafenamide (TAF) should be used, as these are effective against both viruses [2,3,4].

### 2.2. Monitoring of Initial HIV-1 RNA Decay Kinetics

Data on DTG+3TC decay kinetics in real-life contexts are still limited, and our knowledge is mainly based on RCTs. These provided us with reassuring data on the high potency of this combination as an initial regimen, mirrored by the rapid HIV-1 RNA decay kinetics and achievement of undetectable HIV-RNA (TND, target not detected) [8,9], and the low frequency and intensity of viral blips after virological suppression [10,11].

In GEMINI-1 and -2, the median time to reach TND viral load (VL) values with DTG+3TC was comparable to that observed with DTG+emtricitabine/tenofovir disoproxil fumarate (FTC/TDF) arms (8-weeks, median), with no apparent role for higher VL or lower CD4 cell count at BL (Table 3) [7,12,13,14], thus dispelling any doubts that this type of “simplified” therapy could slow down viral decay. Rather, the 117 patients with BL VL >100,000 copies/mL treated with DTG+3TC achieved TND VL values in a median (IQR) time of 16 (16–24) weeks, apparently even shorter than that observed in the 132 receiving DTG+TDF/FTC (median (IQR) = 24 (24–36) weeks) [9] (Table 3). Although interesting, this difference remains not statistically significant, and, therefore, should be evaluated with caution, particularly regarding the lack of related real-life data.

A slow decay-kinetics during initial treatment with DTG+3TC, with persistently positive HIV-RNA through the first 24 weeks of treatment, is an unusual event. In the real-life URBAN study, only 1/96 naïve patients failed to reach the efficacy endpoint of <50 copies/mL at 24-weeks [15]. This patient had an initial VL of 6 log_10_ copies/mL, and experienced a 3.5 log_10_ decay within the 24 weeks of DTG+3TC administration [15].

Even though RCTs represent “controlled settings”, the results obtained in GEMINI-1 and -2 studies are consistent with the real-life experiences we are all witnessing. Therefore, we do not expect an increased risk of incomplete viral suppression with DTG+3TC 2DR (either co-formulated or not) as an initial regimen in patients with characteristics which are compatible with GEMINI’s enrolment, and we do not foresee the need for any early viral decay control following the initiation of treatment.

### 2.3. Assement of Long-Term Treament Efficacy as First-Line Regimen

**Figure 1:**
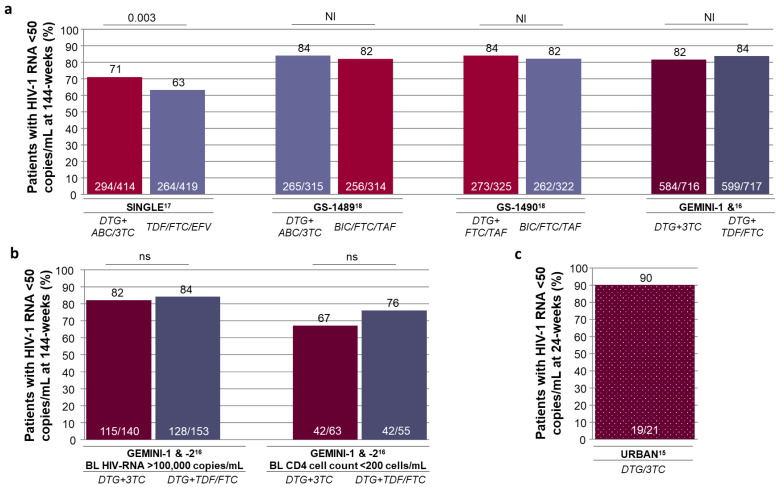
Dolutegravir plus lamivudine two- and three-drug regimens in treatment-naïve patients. (**a**) Efficacy in randomized clinical trials by FDA snapshot analysis at week-144. Histograms represent individual studies—not a head-to-head comparison. (**b**) Efficacy of dolutegravir plus lamivudine in GEMINI-1 and GEMINI-2 randomized clinical trials by FDA snapshot analysis at week-144, in patients with baseline viral load >100,000 copies/mL, and in patients with baseline CD4 cell count <200 copies/mL. (**c**) Efficacy of dolutegravir plus lamivudine in URBAN real-life study, week-24 result in the effectiveness set (excluding missing data). 3TC = lamivudine; ABC = abacavir; BIC = bictegravir; BL = baseline; DTG = dolutegravir; EFV = efavirenz; FTC = emtricitabine; NI = noninferior treatment difference; ns = not statistically significant difference; TAF = tenofovir alafenamide; TDF = tenofovir disoproxil fumarate.

The 3-year follow-up of the GEMINI-1 and -2 studies consolidated the durable non-inferiority of this 2DR vs. DTG+FTC/TDF three-drug regimen (3DR) in cART-naive adults [16]. The long-term efficacy of this 2DR was consistent with the previous results of DTG-based 3DRs in RCTs (Figure 1a) [16,17,18], as was also highlighted by a recent meta-analysis [19]. At 96-weeks of follow-up, virological suppression and CD4 T-cell recovery with DTG+3TC were comparable (if not superior) to those achieved with 3DRs, while witnessing a significant reduction in the frequency of serious adverse events (sAE) [19]. At 144 weeks of follow-up, the primary endpoint of HIV-1 RNA <50 copies/mL was fulfilled by >80% study participants receiving DTG+3TC, and consistently sustained within subgroups for age, sex, race, HIV-subtype, Hepatitis C virus serostatus, and baseline (BL) VL above 100,000 copies/mL (Figure 1b) [16,20]. The rate of viral suppression in patients with a low initial CD4 cell count (<200 cells/mL) receiving DTG+3TC was 67%, vs. 76% of those receiving DTG+TDF/FTC [20]; this difference suggests further analysis, although it is not significant at this point.

The long-term data available today from clinical trials thus support the effectiveness of DTG+3TC 2DR in achieving and maintaining effective virological suppression, comparable to that obtainable with a 3DR.

The emerging real-life data point in the same direction. The URBAN study is a prospective, non-interventional, 3-year German cohort study including naïve patients receiving DTG+3TC or DTG/3TC. At 24-weeks of follow-up, 90% of those with available data had HIV-1 RNA <50 copies/mL (Figure 1c) [15]. The rate of discontinuation through this end-point was very low (<5%), as the safety profile. No sAE was reported, and the most common AE was depression [15].

Altogether, these data support the feasibility of such an approach on a large scale, with no need for substantial pre-treatment diagnostic assessments within the limits of the provided indications for use, and a general confidence in its efficacy, regardless of a number of clinical factors.

### 2.4. Definition of Virological Failure and Resistance Development to Initial Treatment

DTG uniformly shows a high genetic barrier to resistance in both 3DR and 2DR, and resistance-associated mutations (RAMs) are rarely observed. In vitro, DTG-resistance was found to develop much more slowly than for raltegravir (RAL), cabotegravir (CAB) and elvitegravir (EVG) [21], and resistance development during the failure of a DTG-containing 2DR was reported only for isolated clinical cases [16,22,23].

Among drug-naïve patients enrolled in the real-life URBAN study, no virological failures (VF) were observed up to 24 weeks of follow-up [15]. In GEMINI-1 and -2 RCTs, there were low and comparable numbers of participants meeting confirmed virological withdrawals (CVW) criteria through 96 weeks in the DTG+3TC and DTG+TDF/FTC arms [10]. In these patients, no emergent genotypic or phenotypic resistance to INI or NRTIs were observed at 96 weeks [10], and confirmed through 144 weeks [16]. The only exception was one poorly adherent subject in the DTG+3TC arm, who developed the M184V RAM in the reverse transcriptase (RT) at week-132 (HIV-1 RNA = 61,927 copies/mL), and the R263R/K RAM within the integrase (INT) at week-144 (HIV-1 RNA = 135 copies/mL) [16].

Hundreds of thousands of patients worldwide have been given first-line treatment with DTG-containing regimens to date, and this represents the sixth case of failure of a first-line DTG-regimen with emerging mutations (the first occurred in a 2DR). Notably, suboptimal DTG levels were recorded in all of them [16,22]. Emergent mutations included T66I, G118R, Q148R/K, I151V, E157Q, G163E, R263R/K, for the most part associated with a low-level DTG-resistance in vitro [24,25,26]. The copresence of M184V plus R263K was observed in three out of six cases, and this same mutational pattern was also described in a recent case of failure to a bictegravir (BIC)-containing 3DR (M184I/V+R263K, 33% prevalence) [27].

The acquisition of a solitary R263K mutation was shown to confer low-level resistance (~2-fold) to both DTG and BIC in vitro, compatible with the high genetic barrier of the two InSTI [21,25,28,29,30]. In addition, as an R263K-carrying integrase is associated with lower enzymatic-activity in vitro and a decrease in viral-DNA integration [29], its development (and fixation) in vivo likely requires a long-term selective pressure by suboptimal drug levels, or the pre-existence of compensatory mutations, able to counteract its negative impact.

Overall, the development of the same M184V+R263K mutational pattern during treatment with two high-barrier, latest-generation InSTI, requires verification of whether the co-existence of these two RAMs could cooperate in conferring significant InSTI-resistance, while preserving viral infectiousness. In the absence of further data, however, this possibility remains a mere hypothesis.

## 3. Test and Treat Strategy in the COVID-19 Era

In late 2015, WHO eliminated the traditional CD4-based cART eligibility requirements, and recommended treatment for all people living with HIV [31]. Along this line, the “Test & Treat” (T&T) paradigm for rapid initiation of cART—*as early as the day of HIV diagnosis*—or within the following 7 days, was proposed later on [32], mostly in response to the major issue of losses to follow-up in low/middle-income countries [33].

Based on the concept that undetectable virus means untransmissible virus (U = U) [34,35], the “T&T” strategy aims to improve virological control, thereby enhancing the retention-in-care after first diagnosis. The WHO’s “universal test and treat” program has an even wider significance as the strategy for HIV elimination in place of the previous “differed treatment” [32,36].

Although there is no precise definition of time ranges, initiation is considered “rapid” when treatment begins as soon as possible after confirming HIV infection [33]. Rapid-start ART has been adopted by several programmes in low/middle-income countries [37,38,39,40,41], and some high-income countries [42,43,44,45]. Robust evidence demonstrates the benefits of rapid-start strategies, especially in developing countries settings, with a high prevalence of HIV infection, late presentation and limited access to care [38,39,40,46]. Recently, a meta-analysis including 2 RCTs (Haiti and South Africa), 2 cluster-randomized studies (Uganda and Lesotho) and 11 observational studies, showed that same-day cART was associated with a significantly higher proportion of subjects on cART after 90 days, with a higher proportion viral suppression after 12 months, and with a borderline reduction in patients lost to follow-up or dead at 12 months, compared to differed treatment [46].

HIV diagnosis, initial treatment and retention-in-care cannot be compromised by the overwhelming burden of clinical challenges that the COVID-19 pandemic has forced us to face. Bearing in mind that people should not be coerced into starting ART immediately [6], T&T approaches are interesting proposals, by which we could limit the need to access clinics (and doctors) as much as possible, while fully preserving quality care for optimal outcomes.

Under the common aegis of a T&T approach, different real-life modalities are declined: (a) *same-day* treatment, which implies starting cART the same day a final HIV infection diagnosis is made; (b) WHO’s “universal test and treat”, which foresees cART initiation within 7 days from final diagnosis [32]; (c) early-treatment, which allows for cART to start within from 7 to 30 days from diagnosis.

The adoption of a T&T approach implies that the faster the treatment is started, the lower the number of laboratory tests that can be performed and delivered. T&T thus requires a significant revision of the BL virological assessment of HIV patients, and of all those barriers that people encounter before receiving a prescription (e.g., blood drawing, laboratory testing, collecting results, getting an appointment for counseling, and making a decision) [33].

With the current technology, an average microbiology laboratory should be able to provide a confirmed HIV diagnosis, and VL data, within a maximum of 2 days following the first (single) blood withdrawal. Ideally, the antibody/antigen fourth-generation combo formats can be completed within 1–2 h from the sample receipt, and VL measurement technically requires only a few more hours (whether run by automated workstations through a random-access configuration). On the other hand, genotypic resistance test (GRT) results typically require 2–4 weeks to become available. Only a few excellence centres implement fast-track GRT (e.g., for acute infection), while a substantial number of smaller labs require some extra-time, due to the delayed shipment of samples to remote reference laboratories.

In general, the adoption of a T&T strategy would imply starting treatment without genotypic resistance testing (GRT) information, and even without VL data, if a *same-day-treatment* is considered.

### Diagnostic Implications for a Test and Treat Strategy with Dolutegravir Plus Lamivudine Dual-Regimen

The prospect of adopting a simplified 2DR in a T&T approach is intriguing, but not free from criticalities, and needs further validation before it can be applied in clinical practice. In view of using DTG/3TC, major issues concern the possible presence of transmitted RAMs, and the need to assess HBV-serostatus, as 3TC is no longer recommended for the treatment of HBV infection [47] or for HIV/HBV coinfection [2,3,4].

The execution of a baseline GRT is aimed primarily at the exclusion of transmitted NRTI resistance, and, in particular, of the major 3TC RAM M184V. Highly successful clinical trials support the hypothesis of a limited impact (if any) of pre-existing NRTI resistance on virological response to first-line, DTG-based 3DRs [48], and the presence of transmitted M184V/I did not increase the hazard of VF in naïve patients starting a DTG-based cART in clinical practice [49]. At the moment, however, specific data in the context of first-line, DTG-based 2DRs are lacking, owing to the low estimated global prevalence of M184V/I (≤1%) [50], and selective criteria of RCTs [16].

Data on the potential role of transmitted DTG resistance in the context of initial 2DRs are similarly missing, as major InSTI-mutations are found in no more that 1% (usually 0%) of drug-naïve or recently infected individuals included in European studies. A higher proportion of drug-naïve subjects (2% to 17.3%) harbor minor InSTI RAMs, which often occur as polymorphisms [26,51,52,53,54,55,56]. These polymorphisms were likely to exist before InSTI’s introduction into the clinical routine in Europe [57], and are currently considered to have little effect on InSTI susceptibility in vitro, and (most likely) even less in vivo [14,25,48,58,59]. A recent case report reported the successful control of HIV-1 at 24-weeks of DTG/3TC administration in the presence of secondary InSTI RAMs [60].

Overall, the long-term experience with DTG-based 3DRs, along with initial data on 2DRs, sustain the hypothesis that minor transmitted InSTI RAMs are unlikely to play a determining role in globally reducing DTG/3TC 2DR efficacy, whether or not they are associated with major RAMs (an even less likely event). The same concept is applicable to the individual presence of M184V, whose role could be overcome in the context of association with high-genetic barrier drugs, such as DTG [49]. However, although in a completely different clinical context, the still-discussed role of M184V/I in a virologically suppressed patient who switches with DTG/3TC 2DR (see below) requires their possible impact to be taken into consideration, at least at this time.

Due to the lack of specific data, the current international guidelines suggest that DTG/3TC should not be initiated before the results of a genotypic resistance test are available, thereby excluding its use in T&T settings. However, some interesting findings have emerged in recent months.

The STAT study (ClinicalTrials.gov, NCT03945981) is the first phase IIIb, multicenter, open-label, single-arm, pilot study assessing the feasibility, efficacy, and safety of using DTG/3TC as a first-line regimen in a ‘T&T’ approach in primary naïve patients in the US [61]. After the first 24-weeks of treatment, 102/111 (92%) of patients had HIV-1 RNA <50 c/mL at the observed analysis, of whom 87% were receiving DTG/3TC. Through week 24, only a few participants required an adjustment of their initial 2DR regimen (8/131, 6.1%), according to the results provided by BL laboratory testing (1 with transmitted M184V, and 5 with HBV co-infection), or AEs (*N* = 1). None of the included patients had BL InSTI RAMs, so their impact on virological response (and the possibility of adjustments) was not assessable.

Overall, even though the RCT data provide an initial proof-of-concept of the feasibility of using DTG/3TC as a first-line regimen in a T&T setting, the safety of such an approach remains to be further evaluated. Data on transmitted NRTI or InSTI RAMs are reassuring, but mostly obtained from 3DRs. Longer-term studies are thus required to fully assess the impact of either individual or co-present 3TC or DTG RAMs in the contest of initial DTG/3TC 2DR. Until more information becomes available, GRT performance remains an important diagnostic assessment prior to the start treatment. It is thus premature to generalize these RCT data to the highly diversified scenarios seen in clinical practice, and caution regarding transmitted drug resistance and baseline viral coinfections remains mandatory.

## 4. Diagnostic Framework for Dolutegravir Plus Lamivudine Two-Drug Regimens in Virologically Suppressed Patients

With success rates approaching 100%, more and more patients in our clinics are asking us to switch to simpler, more manageable, and less impactful regimens. When it comes to the physician’s decision of whether to switch from a fully effective 3DR to a 2DR or not, the main reason we consider is the possibility of reducing long-term side effects (i.e., a pro-active switch) and, therefore, the frequency of medical visits. In our daily clinical activity, however, we also know that one of the main reasons for the success of 2DR in real-life, especially in single-pill regimens, is the patients’ desire to use an easy, manageable regimen, which has the least impact on their daily routine [15,23,62,63]. This is a need that transversally affects a wide variety (and heterogeneity) of clinical and social conditions, and is felt now more than ever. However, the availability of simpler and more manageable regimens must always be evaluated in the context of a correct diagnostic framing of the patient, and the most appropriate modalities (and timing) of monitoring over time.

### 4.1. Identification of Patients Eligible for Simplified Dolutegravir/Lamivudine Treatment

On August 2020, DTG/3TC 2DR was approved by the FDA as a new single-pill option for people with fully suppressed HIV who wish to replace their current treatment, providing a silent anamnesis for previous treatment failure, or the detection of DTG or 3TC RAMs. Maintaining these indications, DTG/3TC 2DR is currently listed among the recommended regimens for simplifying treatment in virologically suppressed patients in the latest international guidelines, in addition to DTG + rilpivirine (RPV) and boosted protease inhibitor (PI)-based regimens (Table 4).

**Figure 2:**
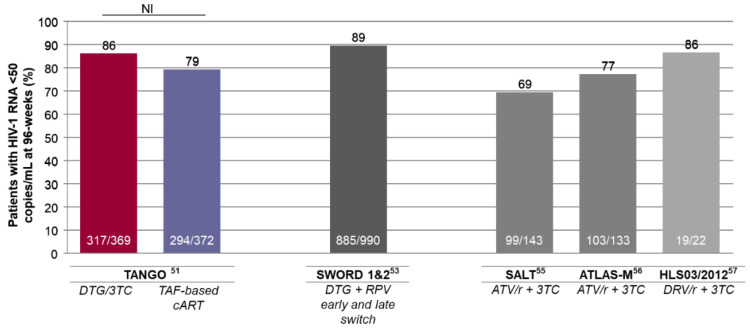
96-week intention-to-treat efficacy of two-drug regimens in virologically suppressed patients in clinical trials. Histograms represent individual studies—not a head-to-head comparison. 3TC = lamivudine; ATV/r = ritonavir-boosted atazanavir; cART = combination antiretroviral therapy; DTG = dolutegravir; DRV/r = ritonavir-boosted darunavir; NI = noninferior treatment difference; RPV = rilpivirine; TAF = tenofovir alafenamide.

The settling basis for these indications comes from the TANGO study, which supported the effective virological suppression at 96 weeks of DTG/3TC combination, non-inferior to tenofovir alafenamide (TAF)-based cART [64,65], in patients without prior virological failure, NRTI resistance, or HBV co-infection (Figure 2).

The 86% rate of virological suppression after 96-weeks of DTG/3TC administration in the TANGO study are in line with the 89% suppression rate observed for DTG plus RPV in the pooled analysis of SWORD-1 and SWORD-2 trials (n/N = 885/990) (Figure 2) [66,67]. It should be noted that, at the same timepoint, the snapshot rates of virological suppression in the patient who switched to atazanavir/r+3TC in SALT and ATLAS-M studies were 69% (99/143) and 77% (103/133), respectively [68,69]. In a recent study, DRV/r+3TC led to a 96-week ITT efficacy of 86% (19/22) (Figure 2) [70]. Even though these are individual studies, and a direct comparison cannot properly be made, the latest IAS guidelines indicates the switch to 2DRs that include a boosted PI (lopinavir, atazanavir, or darunavir) as an option when other NRTIs or dolutegravir cannot be used [3].

From a diagnostic perspective, it is important to point out that the observed efficacy was consistent within several analyzed subgroups, demonstrating a sustained response regardless of age, sex, race, baseline 3rd agent class, and baseline CD4+ cell count [71]. The consistency of results across the various subgroups analyzed toward the overall analysis was also observed in the safety profile [71].

Besides RCTs, real-life studies allowed us to evaluate patient populations that would probably be considered ineligible for a clinical trial, but who represent a significant portion of those treated in our daily practice, as well as those who, probably, would benefit the most from a switch to simpler and better-tolerated therapies.

A recent meta-analysis reported random-effects pooled estimates for a viral suppression of 98.6% and 97.5% at 48-weeks and 96-weeks of DTG/3TC, respectively, by combining the results of 11 real-world studies [72]. In fact, all the real-life studies available today, including the most recent, report on the excellent and sustained efficacy of this 2DR when used as a switch regimen (Table 5) [15,23,62,73,74,75,76,77,78,79,80,81,82,83,84]. These results are consistently maintained in both patients with a previous history of VF and resistance [23,62,74,75,76,80,82], and over longer observation periods [75,76,84]. Over a 4-year follow-up, the most recent DTG/3TC results in a real-life prospective cohort of 218 treatment-experienced patients reported a 100% virological-efficacy, with no VFs [84]. From the analysis of 8 years of the use of dual therapies in simplified regimens, including DTG/3TC, an Italian multicenter study showed that DTG-based 2DR does not increase the risk of viral rebound (n = 157 subjects) as compared to cART (2NRTIs plus a boosted-PI or DTG, n = 516 subjects), even in a real-life clinical context [63].

Overall, this evidence in support of the effectiveness of the strategy of simplification to DTG/3TC identifies, and also validates a simple diagnostic procedure to frame the switch in the eligible patients, which is actually limited to a few characteristics (resistance, coinfection with HBV). In this regard, it has to be underlined that patients with HBV co-infection should continue taking TAF or TDF (unless contraindicated), as switching to a regimen not including these drugs fails to maintain HBV suppression [2,3,4].

### 4.2. Diagnostic Definition of Virological Failure and Evaluation of Resistance Development after Switch

Virological failure in RCTs is usually defined as one measurement of HIV-1 RNA ≥50 copies/mL, followed by a confirmatory measurement of HIV-1 RNA ≥200 copies/mL. This eventuality leads to the need to perform a genotypic resistance test, by either Sanger or ultra-deep sequencing, to assess the development of resistance mutations.

DTG in dual-formulations has a high genetic barrier to resistance development, as evidenced by the extreme rarity of CVW from long-term RCTs [64], as well as VFs in real-life studies (Table 5) [15,23,62,73,74,75,76,77,78,79,80,81,82,83,84].

In the 96 weeks of the TANGO study, no CVWs were observed, not even in patients who contracted COVID-19 [64]. Likewise, virological failures leading to DTG/3TC discontinuations were rare across all real-life studies, and no treatment-emergent DTG resistance was discovered, even though GRTs at failure were not always available (Table 5). In only one case, a new NRTI mutation (M41M/L, mostly driven by treatments with thymidine analogues) appeared at failure of the 2DR with DTG+3TC [23], yet with an uncertain role (if any) in the occurrence of failure itself (transmitted mutation in a minority form? Previous treatments with thymidine analogues?). Long-term data from a prospective cohort study recently reported that, over a 4-year observation period, the main reasons for DTG/3TC discontinuation were related to the worsening of co-existing comorbidities, rather than VF (or drug-related AEs) [84].

Despite these results, the GRT at failure remains an indispensable tool of clinical evaluation, in order to better identify the next therapeutic strategy, and, as such, is strongly recommended.

## 5. Diagnostic Protocols for HIV-1 Residual Viremia and Reservoir Monitoring

An increasing amount of evidence from real-life experiences and clinical trials consistently contribute to the indication of a negligible risk of incomplete control of viral replication, either in terms of the enrichment of HIV-DNA reservoir, residual low-level viremia, or viral rebound/blips, in patients treated with DTG/3TC 2DR vs. standard cART, thus avoiding the need for more stringent monitoring of virological suppression than with 3DR, once the dual regimen is established.

**Figure 3:**
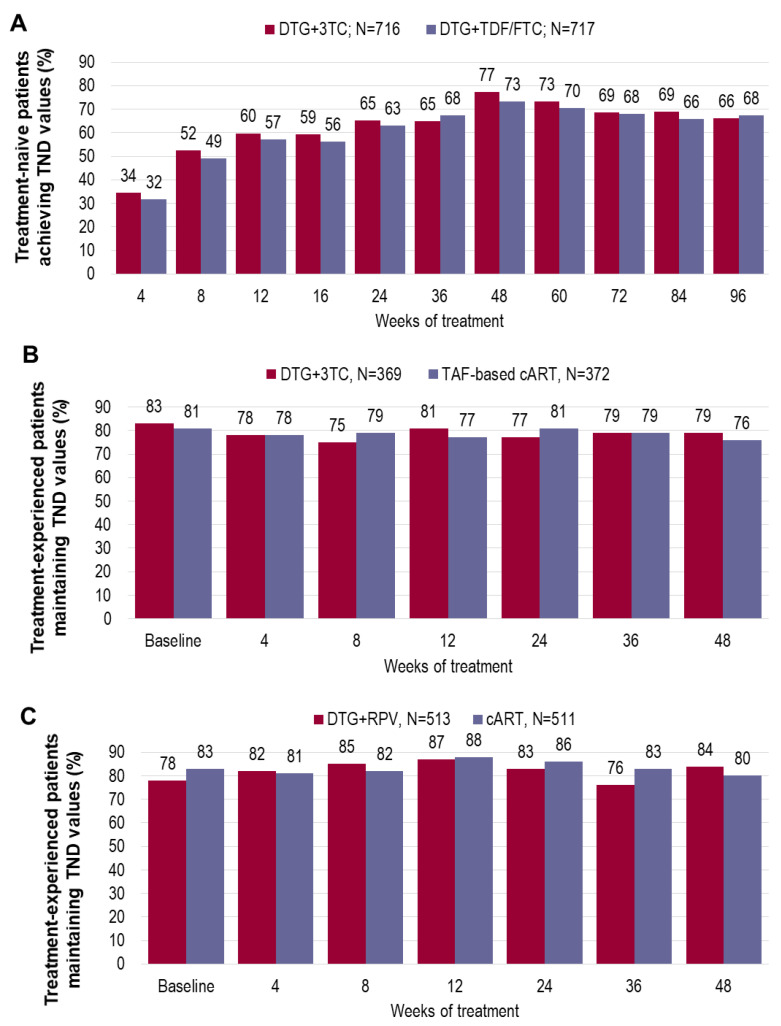
Achievement and maintenance of non-detectable viral-load values in patients enrolled in dolutegravir-based two-drug regimens clinical trials. Histograms represent individual studies—not a head-to-head comparison. (**a**) Proportion of drug-naïve participants with TND values by week of treatment from GEMINI-1 and -2 studies (snapshot analysis). (**b**) Proportion of treatment-experienced participants with TND values by week of treatment from the TANGO study (snapshot analysis). (**c**) Proportion of TND values by week of treatment for treatment-experienced participants with TND at baseline, from the SWORD-1 and SWORD-2 studies. 3TC = lamivudine; cART = combination antiretroviral therapy; DTG = dolutegravir; FTC = emtricitabine; RPV = rilpivirine; TAF = tenofovir alafenamide; TDF = tenofovir disoproxil fumarate; TND = Target not detected.

In GEMINI-1 and -2 studies, the number of participants reaching TND values at each week visit was similar to DTG+3TC and 3DR, up to the latest follow-up (96-weeks; Figure 3a) [9], supporting the persistent control of low-level VL when DTG+3TC is used as an initial regimen. Furthermore, this 2DR has been shown to have a “regimen forgiveness” (defined by the ability to preserve virological suppression in situations of suboptimal adherence), comparable to that of DTG+TDF/FTC 3DR in a post-hoc pooled analysis of the GEMINI-1 and -2 studies at 48-weeks [85]. None of the 18 CVWs in GEMINI-1 and -2 RCTs had VL blips that preceded CVW [10,11].

Consistently, no modifications in the standard follow-up schedule are foreseen for treatment-experienced patients. A recent real-life study reported the excellent efficacy of DTG/3TC in maintaining a steady virological control of residual viremia, below the limit of 50 copies/mL, over 48 months of observation [84]. Viral blips appear to be extremely sporadic and are usually below the 200 copies/mL [84], mirroring previous results from clinical trials. In the TANGO study, there was no difference in the proportion of participants with VL <40 copies/mL or TND through 48-weeks [65], and the snapshot efficacy of DTG/3TC 2DR was confirmed by using these more stringent definitions of viral suppression [65]. The comparable proportion of patients with TND values among the 2DR and 3DR arms in the TANGO studies (Figure 3b) [65] retraces the results obtained with the DTG+RPV combination in the SWORD-1 and -2 studies, where DTG+RPV 2DR and standard 3DR guaranteed the same maintenance rate of complete virological suppression at each study-visit (Figure 3c) [11].

The quantification of total HIV-DNA in peripheral blood mononuclear cells (PBMCs) represents the most widely used indicator of the HIV cellular reservoir size and dynamics [86]. Both MONI [87] and MONET trials [88] provided initial data on the possibility to maintain the control of HIV-1 DNA levels through 144-weeks by using DTG monotherapy vs. continuation of triple therapy, a proof-of-concept supported by the stability of HIV-DNA levels observed in the pilot DTG monotherapy MONODO study [89]. These results need to be considered only as proof-of concepts, since monotherapy with DTG is not recommended by any guidelines.

Although still poorly studied, the available data suggest similar dynamics of total HIV-DNA decay in DTG/3TC and in patients treated with cART up to 48 weeks post-switch [90], and thus an effective control of viral reservoir by simplified regimens. A counterproof of this hypothesis is provided by the Be-OnE Study. This randomized, single-center, open-label, superiority study has recently compared the maintenance of virological suppression in a cohort of 40 virologically suppressed individuals while receiving DTG plus one NRTI for at least 3 months, randomized 1:1, to continue the ongoing treatment or switch to EVG/c/FTC/TAF. In those who switched to 3DR, changes in HIV-DNA and residual viremia from baseline to 48 weeks were negligible, and did not significantly differ from changes in those who continued DTG-based 2DR [91], supporting the non-inferiority of DTG-based 2DR vs. EVG/c/FTC/TAF in the control of viral reservoir.

Overall, the increasing amount of evidence from real-life experiences and clinical trials consistently contribute to the indication of a negligible risk of incomplete control of viral replication, either in terms of the enrichment of an HIV-DNA reservoir, residual low-level viremia, or viral rebound/blips, in patients who either start on, or switch to, a DTG/3TC 2DR vs. standard cART, therefore supporting the use of diagnostic follow-up protocols with similar timelines to those currently in use.

While the data are quite consistent, it should be emphasized that the currently available real-life studies include patients with a long history of virological suppression (years), and, in a relevant percentage, switched while already on other 2DRs. Therefore, further studies are warranted.

## 6. Assessment of Pre-Existing or Archived Resistance

Some real-life studies have agreed with the RCTs in excluding patients who presented InSTI or NRTI RAMs at BL, or who had previously experienced a VF. A small number of studies, however, used less stringent enrollment criteria, and allowed us to analyze the behavior of DTG/3TC in the presence of RAMs, and/or in patients with an history of failures [23,62,74,75,77,80,82,92,93,94,95,96] (Table 5).

Given the rarity of DTG RAMs, the BL resistance (in historical or contextual GRTs) is usually defined by the presence of the NRTI RAMs M184I/V.

The MOBIDIP study provided the initial proof-of-concept that a 2DR including 3TC could retain partial efficacy, despite HIV-infected patients harboring a 3TC-resistant virus (i.e., with M184V in their historical genotype) [97]. The study was prematurely discontinued after 48 weeks for the exceeding proportion of VFs in patients receiving boosted-PI monotherapy vs. 3TC+boosted-PI [97], but the hypothesis of the retained activity of 3TC in presence of M184I/V, whether used within potent 2DR, was maintained.

On the other hand, an initial warning was launched by two real-life studies, which observed an increased risk of VF associated with the presence of M184V [75,93]. In one of these studies, the risk was limited to patients with a shorter virological suppression time before the switch (<96 months), albeit at the limit of statistical significance [75]. All other real-life data available today, however, agree in terms of the exclusion of an association between the M184V mutations in historical GRTs and VF to DTG/3TC, at least up to 48- or 96-weeks after switch [23,74,75,77,80,92,94,95,96], and regardless of the duration of the time-lapse between the detection of RAMs and the start of 2DR [23].

An interesting in vitro study has advanced the hypothesis that the NRTI RAMs K65R, M184I and M184V may have a protective role in the context of DTG-based regimens (but not with raltegravir or elvitegravir), antagonizing the development of specific resistance [98]. This phenomenon may cooperate with the possible residual activity of 3TC in the presence of M184V/I [97], and the presence of other contingent factors (i.e., high adherence, long duration of pre-switch virological suppression) in providing an explanation for the rarity of DTG/3TC failure in the presence of BL NRTI RAMs, as well as the lack of InSTI resistance development in those few cases. This hypothesis requires confirmation and an in-depth evaluation before being considered clinically relevant.

### Added Value of Proviral DNA Testing in Clinical Settings

It is still unknown whether proviral DNA genotyping in virologically suppressed patients could contribute to a prediction of the efficacy of 2DRs, and if it would add significant information to the historical genotype. In the TANGO study, the archived major NRTI RAMs (e.g., M184V/I, K65N/R, and TAMs) and INSTI RAMs (e.g., Q148R, Y143C/H, R263K) were identified in a minority of patients on pro-viral DNA (1% and 7%, respectively) [99]. Their minoritary presence (1–7%) did not increase the risk of intermittent viremia through week-48 [100], or affect treatment outcomes at 48 weeks, as viral suppression was maintained in >99% of participants receiving DTG/3TC or TAF-based 3DR [99]. Similar results were obtained in the ART-PRO real-life, pilot trial, where the M184V/I and/or K65R/E/N mutations in pro-viral DNA did not affect virological outcome at 48 weeks [80]. No cases of withdrawal due to virologic failure, or emergence of resistance were observed, not even in the 21 subjects with M184I/V above the 5% threshold in pro-viral DNA [80].

It should be noted that the detection of the M184I in pro-viral DNA is known to be associated with the hypermutation activity of the apolipoprotein B mRNA editing enzyme, catalytic polypeptide 3G/F (APOBEC3G/F) [101,102]. As the amino acid substitutions generated through APOBEC3G/F hypermutation are not associated with an increased risk of virological failure to first-line regimens [101], the finding of M184I in pro-viral DNA is unlikely to represent a possible determinant of resistance.

Overall, with due caution, the data available today seem to exclude the hypothesis that patients with an underlying presence of M184V/I could find themselves in a condition of functional DTG monotherapy (especially if these RAMs are archived). These initial, encouraging data lead us to believe that, on the one hand, the clinical GRT interpretation must be differently calibrated (and reinterpreted) in the context of the switch to highly potent 2DRs. On the other hand, however, there are still many relevant aspects of the mutational virological profile that have not sufficiently declined in terms of their possible impact. Among these, InSTI RAMs could represent a critical element in the future, although this is currently rare. Similarly, the possible correlation between the (higher) number of previous virological failures with a complex resistance pattern, and the viraemic rebound with DTG/3TC is a still largely unexplored aspect, with few data recorded in the literature. These considerations, therefore, do not spare us, in clinical practice, from a carefully evaluation of the clinical history, baseline virological parameters, and historical GRT of virologically suppressed patients in the process of switching to a 2DR.

The added value of pro-viral DNA testing in clinical settings is somewhat controversial, and not yet defined. According to international guidelines (e.g., DHHS), drug resistance results on pro-viral DNA must be interpreted with caution [2]. The main criticalities lie in the lack of standardization, and in the possible failure in identifying all previously existing drug-resistance mutations (including M184V) [103,104,105]. Furthermore, the pro-viral DNA does not necessarily correspond to a functional reservoir, and the mutations found may belong to a defective DNA, or derive from hypermutation phenomena [101,102]. Significant progress in this area depends on the availability and widened use of standardized HIV-DNA assays, though the data obtained to date represent a reasonable starting point to evaluate the role of HIV-DNA testing in clinical practice.

## 7. Monitoring of Treatment Safety

Real-life data consistently report an overall excellent tolerability of DTG/3TC, with an absence/low incidence of toxicity [15,62,73,76,78,81,106,107], and improvements in CD4 count [62,73,76,78], CD4:CD8 ratio [62,74,78,107], and lipid profile [62,73,74,76,77,78], when DTG/3TC is used as initial or switch strategy. Patients begin to report significant improvements in symptom distress and treatment satisfaction after the first few months of initial treatment [15]. In treatment-experienced patients, DTG was independently associated with a lower discontinuation risk, as compared with both darunavir/r- and atazanavir/r [62]. A meta-analysis of 11 studies, including virologically suppressed subjects treated with DTG+3TC, estimated a rate of discontinuations of 10.4% at 48 weeks, and of 11.8% at 96 weeks [72]. At 4 years of follow-up, 80.7% of subjects included in a multicenter cohort of patients switched to DTG/3TC are still effectively treated with this 2DR, and most discontinuations depended on underlying pathologies, and not on AEs attributable to the antiviral treatment [84]. This supports the sustainability of this switch regimen, even in real-life cohorts, which are often particularly enriched by subjects with multiple comorbidities and problems related to old age.

The achievement of a long follow-up in the RCTs important data to be obtained in support of the safety of the DTG plus 3TC regimen, whether it is co-formulated or not. In the 144 weeks of GEMINI-1 and -2 studies, participants in the DTG+3TC group had a significantly lower risk of drug-related AEs (20%) compared with the DTG+TDF/FTC group for the pooled population (27%; relative risk, 0.76) [16]. In treatment-experienced patients, the frequency of AEs substantially overlapped between DTG/3TC and TAF-based cART over 96 weeks across various subgroups, and discontinuation due to AEs was extremely rare [64,71].

Patients with impaired renal function raise significant issues for cART tolerability, and this type of toxicity is one of the main reasons leading to therapeutic switch, especially from TDF-based regimens. RCTs have demonstrated a good renal safety profile of DTG/3TC in drug-naïve patients, superior to that observed with DTG+TDF/FTC at week-144 [16], a result also confirmed in virologically suppressed patients, in whom DTG/3TC maintained the level of safety guaranteed by a TAF-based cART [64]. Through the 96-weeks of TANGO study, the decrease in eGFR by cystatin C was significantly lower with DTG/3TC than with TAF-based cART. Some real-life studies have observed a reduction in eGFR following the switch to DTG-based 2DRs, probably due to the inhibition of organic cation transporter 2 by DTG [81].

Interestingly, a recent case review reported no virologic failure in 25 patients who switched to DTG/dose-adjusted 3TC once daily, over a median time of 2.5 years of observation [106]. This proof-of-concept contributes to the provision of further confidence that simplification to once-a-day therapy with DTG and dose-adjusted 3TC may, in fact, be a viable solution in those patients with compromised renal functionality.

## 8. Conclusions

The outbreak of the COVID-19 pandemic has created significant challenges for healthcare systems across the world and has extensively affected the life of all people living with HIV. However, HIV diagnosis, initial treatment and retention-in-care cannot be compromised. This situation calls for the adoption of regimens with: (a) high barrier to resistance (to reduce the frequency of monitoring); (b) favorable safety profile and few major drug–drug interactions (to reduce the frequency of access to clinical services); (c) preferentially in a single tablet without food requirements (to improve adherence and retention in care) [1]. HIV-infected patients should continue to receive quality care for optimal outcomes, and the possibility of using simplified 2DRs with such characteristics needs special consideration. However, once we tend to simplify the therapeutic approach, it is necessary to implement the diagnostic part. This should not be intended as the introduction of new diagnostic tests, but a better structure of the diagnostics, once the therapeutic approach is different. The aim of this review was to revise the most relevant diagnostic aspects in the context of dual-therapies, identifying the unavoidable aspects from those that can reasonably be avoided, postponed, or changed to less dramatic contingent situations. Indeed, the clinical, socioeconomic, and psychological impact of the pandemic should not affect the long-term care of people living with HIV, which creates an urgent need to optimize the diagnostic approach to first-line or switch treatments. The possibility of using DTG/3TC 2DR represents an outstanding tool, whose expected advantages fulfill the current requirement for the optimal daily care of HIV patients.

## Figures and Tables

**Table 1:** Latest international recommendations for two-drug regimens in cART-naïve patients.

Regimen	DHHS 2021 [2]	IAS-USA 2020 [3]	EACS 2020 [4]
**DTG+3TC**	Recommended [AI] ^a^ VL < 500,000 HBsAg negative	Recommended [AIa] ^d^ VL < 500,000 CD4 > 200 (perhaps) HBsAg negative	Recommended VL < 500,000 HBsAg negative
**DRV/r or DRV/c + RAL**	Alternative [CI] ^b^ VL < 100,000 CD4 > 200		Alternative ^b^ HBsAg negative VL < 100,000 copies/mL CD4 > 200 cells/μL With food
**DRV/r or DRV/c + 3TC**	Alternative [CI] ^b,c^		

^a^ Except for individuals with HIV RNA > 500,000 copies/mL, HBV co-infection, or in whom cART is to be started before the results of HIV genotypic resistance testing for reverse transcriptase or HBV testing are available. ^b^ When the use of tenofovir disoproxil, tenofovir alafenamide, or abacavir is contraindicated or not desirable; ^c^ 3TC may be substituted for entricitabine, or vice versa; ^d^ Except for individuals with HIV RNA >500,000 copies/mL, HBV co-infection, currently treated for an active opportunistic infection, or in whom cART is to be started before the results of baseline laboratory evaluation. Exclusion of patients with CD4 cell count below 200/μL is unclear. 3TC = lamivudine; DRV/r = darunavir/ritonavir; DTG = dolutegravir; EACS = European AIDS Clinical Society; DHHS = U.S. Department of Health and Human Services; IAS = International AIDS Society; LPV/r = lopinavir/ritonavir; RAL = raltegravir; VL = viral load.

**Table 2:** Laboratory testing for people with HIV before and after initiation of initial antiretroviral therapy.

Laboratory Test	Prior of Starting DTG+3TC	Follow-up Frequency
**Immunology**		
CD4 absolute count and % CD4/CD8 ratio CD8 and % (optional)	Yes	3–6 months ^a^
**Virology**		
Plasma HIV viral load	Yes	3–6 months
HIV genotypic resistance testing and sub-type	Yes	In case of virolgical failure
**Co-Infections**		
Hepatitis B serology ^b^	Yes	Annual/if indicated ^c^
Hepatitis C screening	No ^d^	Annual/if indicated ^e^
Sexually transmitted infections	No ^d^	Annual/if indicated
Tuberculosis	No ^d^	In case of exposure
**Biochemistry**		
Basic chemistry	Yes	6 months
ALT, AST, total bilirubin	Yes	6 months
Complete blood count with differential	Yes	3–12 months
Serum glucose and fasting lipids	Yes	Annual
Urinalysis	Yes	6 months
**Pregnancy test**	Yes	

^a^ After the first 2 years, if virologically suppressed and CD4 count >350 cells/μL, the frequency of monitoring could be annual. ^b^ If HBsAg, HBsAb, and HBcAb test results are negative, hepatitis B vaccine series should be administered. Consider performing an HBV viral load test for confirmation, to account for HBsAb loss. ^c^ If patient is nonimmune and does not have chronic HBV infection; ^d^ Not indicated, if performed at time of HIV-infection diagnosis. ^e^ Repeat HCV screening every 12 months for at-risk patients. ALT = alanine aminotransferase; cART = combined antiretroviral therapy; AST = aspartate aminotransferase; CD4 = CD4 T lymphocyte; HBcAb = hepatitis B core antibody; HBsAb = hepatitis B surface antibody; HBsAg = hepatitis B surface antigen; HBV = hepatitis B virus; HCV = hepatitis C virus.

**Table 3:** Weeks to virological-suppression across treatment groups and baseline strata in observed analysis from GEMINI-1 and -2 clinical trials.

Observed Analysis at 96-Weeks ^a^		Weeks to HIV-1 RNA <40 Copies/mL and Target Not Detected, Median	95% Confidence Intervals
DTG+3TC, *N* = 616		8	NE, NE
DTG+TDF/FTC, *N* = 642		8	NE, NE
Baseline subgroups			
VL ≤ 100,000 c/mL	DTG+3TC, *N* = 499	8	(~8, ~8)
	DTG+TDF/FTC, *N* = 510	8	(~8, ~8)
VL > 100,000 c/mL	DTG+3TC, *N* = 117	16	(~16, ~24)
	DTG+TDF/FTC, *N* = 132	24	(~24, ~36)
CD4+ cells >200 cells/mm^3^	DTG+3TC, *N* = 573	8	(NE, NE)
	DTG+TDF/FTC, *N* = 594	8	(NE, NE)
CD4+ cells ≤200 cells/mm^3^	DTG+3TC, *N* = 43	16	(~12, ~24)
	DTG+TDF/FTC, *N* = 48	12	(~8, ~24)

^a^ Observed analysis includes subjects virologically suppressed at 96-weeks, and thereby censors earlier failures including those unrelated to efficacy. 3TC = lamivudine; DTG = dolutegravir; FTC = emtricitabine; NE = not evaluable; TDF = tenofovir disoproxil fumarate; VL = viral load.

**Table 4:** Latest international recommendations for the switch to oral two-drug regimens in patients who achieved virological control with three-drug cART.

Regimen	DHHS 2021 [2]	IAS-USA 2020 [3]	EACS 2020 [4]
**DTG+RPV**	Recommended [AI] ^a^	Recommended [AIa] ^c^	Recommended ^e^
**DTG+3TC**	Recommended [AI] ^a^	Recommended [AIa] ^c^	Recommended ^e^
**DRV/r+3TC**	Alternative [BI] ^b^	Alternative [AIa] ^d^	Recommended ^e^
**ATV/r+3TC**	Alternative [CI] ^b^	Alternative [AIa] ^d^	Recommended ^e^
**LPV/r+3TC**	Alternative [CI] ^b^	Alternative [AIa] ^d^	-
**DRV/r+RPV**	-	-	Recommended [supported only by small trials] ^f^
**DRV/r+DTG**	Alternative [CI]	-	Recommended [supported only by small trials] ^f^

^a^ In the absence of documented drug-resistance and HBV co-infection. ^b^ When the use of tenofovir disoproxil, tenofovir alafenamide, or abacavir is contraindicated or not desirable. ^c^ In patients with no prior virological failure or drug resistance, and no HBV co-infection. ^d^ When other NRTIs or dolutegravir cannot be used. ^e^ In patients with no prior virological failure or drug resistance. ^f^ Only to persons with (a) no historical resistance, (b) suppression of HIV viral load to <50 copies/mL for at least the past 6 months and (c) absence of chronic HBV co-infection. ATV/r = atazanavir/ritonavir; cART = combination antiretroviral therapy; DRV/r = darunavir/ritonavir; DTG = dolutegravir; EACS = European AIDS Clinical Society; DHHS = U.S. Department of Health and Human Services; IAS = International AIDS Society; LPV/r = lopinavir/ritonavir; RPV = rilpivirine; VL = viral load. The strength of recommendations is reported as in each guideline.

**Table 5:** Real-life experiences of switch to dolutegravir/lamivudine two-drug regimens in virologically suppressed HIV-1 patients.

Study	Design	SubjectsN	Previous Failures Allowed	Latest Follow-up (Weeks)	HIV-RNA <50 copies/mL (%)	No. of VF ^e^	Emerging RAMs ^g^	Baseline M184V/I Role in Failure
MAGGIOLO, 2017 [73]	Prospective, multicenter, cohort study	94	Yes ^a^	24W	100% (ITTe)	0		No role
BORGETTI, 2018 [74]	Retrospective, single-centre, cohort study	206	Yes	96W	80.5% ^b^	4 ^f^	-	-
BALDIN, 2019 [75,76]	Retrospective, single-centre, cohort study	221	Yes	144W	95.3% ^b^	5 ^f^	No	-
BORGHETTI, 2019 [62]	Retrospective, single-centre, cohort study	183	Yes	96W	92.6% ^b^	3 ^f^	-	-
CICCULLO, 2019 [77]	Retrospective, multicenter, cohort study	229	Yes	96W	95.3% ^b^	6 ^f^	No	-
DOLAMA [78]	Retrospective, multicenter, cohort study	177	No	48W	83.6% (ITTe), 97% (PP)	5	No	Yes ^h^
LOMBARDI, 2019 [79]	Retrospective, multicenter, cohort study	67	Yes	48W	100% (PP)	0		No role
ART-PRO [80]	Non-randomized, single-arm, pilot phase IIa clinical trial	41	Yes	48W	92.7% (ITTe), 100% (PP)	0		No role
BALDIN, 2020 [81]	Retrospective, multicenter, cohort study	350	Yes	24W	95.5% ^c^	1	-	-
BATTAGIN, 2020 [82]	Retrospective, single-centre, cohort study	61	Yes	24W	96.7% (ITTe)	2	-	No role
GAGLIARDINI, 2020 [83]	Retrospective, multicenter, cohort study	966	Yes	96W	76.8% ^b^	18^f^	-	No role
GALIZZI, 2020 [23]	Retrospective, single-centre, cohort study	307	Yes	96W	92.9% ^b^	17 ^f^	NRTI: M41L (*N* = 1)	No role
MAGGIOLO, 2020 [84]	Prospective, multicenter, cohort study	218	No ^a^	192W	100% ^b^	0		No role
URBAN [15]	Prospective, multicenter cohort study	282	Yes	24W	94% ^d^	2	No	-

^a^ Patients were not included if they had a viral failure following their last genotypic test. ^b^ Proportion of patients free from virological failure. ^c^ Probability of maintaining the study regimen. ^d^ effectiveness set (missing = excluded). ^e^ Leading to dual-therapy discontinuation. ^f^ Calculated on overall person-years of follow-up. ^g^ Only patients with available baseline and failure genotypic resistance tests. ^h^ Reduced efficacy only in patients with prior viral suppression <96 months. “-” = information not available; ITTe = intention-to-treat exposed analysis; NRTI = nucleoside analog reverse-transcriptase inhibitor; PP = per-protocol analysis; RAMs = resistance-associated mutations; VF = virological failure.

## Data Availability

Not applicable.
